# Policy gap: most child-appealing packaged food products in Canada will display a ‘high in’ front-of-package nutrition symbol

**DOI:** 10.1017/S1368980025101535

**Published:** 2025-12-05

**Authors:** Hayun Jeong, Christine Mulligan, Laura Vergeer, Jennifer J. Lee, Mary R. L’Abbe

**Affiliations:** 1 Department of Nutritional Sciences, Temerty Faculty of Medicine, University of Toronto, Toronto, ON M5S 3H2, Canada; 2 School of Nutrition, Faculty of Community Services, Toronto Metropolitan University, Toronto, ON, Canada

**Keywords:** Front-of-package labelling, Marketing to children, Child health, Public health, Food environment

## Abstract

**Objective:**

Canadian front-of-package (FOP) labelling regulations aim to improve dietary patterns by identifying foods high in sodium, sugars and/or saturated fat with a ‘high in’ FOP nutrition symbol. However, child-appealing marketing on product packaging may undermine these efforts. Therefore, this study (1) compared the prevalence of FOP symbols between products with child-appealing and non-child appealing packaging in the Canadian food supply and (2) identified the number and types of FOP symbols on products with child-appealing packaging (CAP).

**Design:**

Using the University of Toronto’s Food Label Information and Price 2017 database, 5850 packaged foods were analysed, 746 of which had CAP. Products were assessed against FOP labelling regulations.

**Setting:**

Large grocery retailers by market share in Canada.

**Participants:**

Foods and beverages available in 2017. Results: 74·4 % of products with CAP would require a ‘high in’ FOP symbol, significantly higher than the 65·7 % of products with non-CAP. Notably, 54·4 % of products with CAP exceeded FOP labelling thresholds for sugars compared with 37·8 % of products with non-CAP.

**Conclusions:**

Findings highlight a policy gap in Canadian nutrition regulations, as CAP remains a major source of marketing of unhealthy foods to children, undermining the impact of FOP labelling. To address this, food packaging should be included in Canada’s marketing restrictions, and products displaying a ‘high in’ FOP symbol should be automatically restricted from marketing to children. This study underscores the urgent need to harmonise Canadian nutrition regulations to synergistically promote healthier food choices among children and improve their health.

The increasing global prevalence of childhood obesity, which has surged from 2 % in 1990 to 8 % in 2022 and is projected to double by 2035, significantly raises the risk of premature death and disability in adulthood^(1–3)^ and is closely linked to poor diet quality^(1,4)^. National survey data show that Canadian children’s diets are high in saturated fat, free sugars and sodium^(5)^, which have been identified as nutrients of public health concern (‘nutrients-of-concern’ hereinafter), given their associations with increased risk for obesity and diet-related chronic diseases^(6,7)^. Front-of-package (FOP) labelling, a simple and easy-to-understand symbol displayed on the front of food and beverage packages to communicate the healthfulness of the food^(8)^, is a public health tool for improving dietary patterns on a population level^(8,9)^. FOP labelling has been shown to effectively improve consumers’ food choices by communicating nutritional information in a simple and interpretive way to help consumers easily and correctly identify the healthfulness of foods^(8,10)^. FOP labelling can also improve the nutritional quality of the food supply by incentivising manufacturer-driven product reformulation^(9,11)^. In July 2022, Canada’s FOP labelling regulations were finalised as part of the Healthy Eating Strategy with the primary goal of making the healthier choice the easier choice for people living in Canada^(12)^. The Canadian FOP labelling system is nutrient-specific^(8)^, identifying pre-packaged food and beverage products meeting and/or exceeding thresholds for nutrients-of-concern (i.e. saturated fat, total sugars and sodium) with a ‘high in’ nutrition symbol on the front of the food package by January 2026^(12)^. However, unlike the Chilean and Mexican Food Labelling and Advertising Law among other countries in South America, that prohibits child-appealing marketing on product packaging for those that would display ‘high in’ FOP warning labels^(13)^, the Canadian FOP labelling regulations do not encompass any marketing restrictions, including marketing to children.

With research suggesting that people with lower nutrition literacy benefit more from having FOP labelling to guide them in identifying healthier and less healthy foods^(14)^, the potential of FOP labelling to improve the food choices and diets of children who are still learning and acquiring nutrition literacy is promising^(8)^. However, despite the presence of FOP labelling, children will still face an immense barrier to healthy eating as they are targeted by frequent and powerful marketing of unhealthy foods and beverages^(15,16)^. Children in Canada are exposed to a substantial amount of unhealthy food and beverage marketing every day^(17)^ across digital media channels, at schools and at point-of-sale in grocery stores and restaurants^(18)^. The World Health Organization (WHO) identifies unhealthy food marketing to children (‘Marketing to Kids (M2K)’ hereinafter) as a threat to public health^(19)^, given its overwhelming prevalence coupled with children’s limited ability to understand the persuasive power of advertising negatively affects their food preferences and choices, and consequently, their overall diet quality^(19,20)^.

Product packaging has been identified as a top source of children’s exposure to M2K^(21)^. Manufacturers strategically use marketing techniques such as the use of characters, games and other child-appealing graphic designs (e.g. unusual colours and shapes) to influence children’s food choices^(19,22)^. Nevertheless, product packaging has been excluded from Health Canada’s proposed regulations to restrict M2K to children under 13 years of age^(23,24)^. This is concerning given close to 13 % of products (> 700) in a representative sample of packaged foods and beverages from Canadian grocery stores were found to be marketed to kids using child-appealing content on their packaging^(22)^ and were higher in total sugars and free sugars than products with non-child-appealing packaging (CAP). While these prevalence and nutrient findings have been previously reported^(22)^, the present study addresses a policy-relevant concern: if the proposed marketing regulations are passed as is, products identified as unhealthy by FOP labelling may still have CAP that could undermine the effects of FOP labelling on children. There is currently no study to date that has examined this policy gap between FOP labelling regulations and the presence of CAP, as well as the potential challenges that may arise. Unlike other countries such as Chile and Mexico^(13)^, Canada’s FOP labelling regulations do not prohibit foods with FOP symbols from being M2K, nor do Canada’s proposed M2K regulations include product packaging. The objectives of this study were therefore to (1) compare the proportion of products that would display an FOP symbol between products with CAP and non-CAP and (2) determine the number and types of FOP symbols that would be displayed on products with CAP.

## Methods

This study was a cross-sectional analysis of University of Toronto’s Food Label Information and Price (FLIP) 2017 database, described in detail elsewhere^(25)^. Briefly, FLIP 2017 contains package and price information for over 17 000 branded packaged foods and beverages from top Canadian grocery retailers, representing 68 % of Canada’s 2017 grocery retail market share. FLIP 2017 contains data from a product’s Nutrition Facts table (NFt) and ingredients list, as well as photos of all sides of the product packaging, among other information. Although more recent collections exist (e.g. FLIP 2020), FLIP 2017 was used because all subsequent iterations were collected via web scraping due to COVID-19 restrictions on in-store data collection and therefore lack complete images of all package sides, particularly the back of packages, where child-appealing features such as games are often displayed. This makes FLIP 2017 the most suitable dataset for accurately identifying CAP. All products in FLIP are categorised according to Health Canada’s Table of Reference Amounts for Foods (TRA) major and sub food categories, which are used in nutrition labelling regulations to determine typical amounts consumed in a single setting^(26)^.

The analytic sample for this study was derived by selecting a subset of priority TRA subcategories for analyses of child-appealing marketing, as determined through prior evaluations^(27–29)^. Subcategories making up the top 85 % of child-appealing packages were selected (e.g. cookies, cereals and ice cream), and any additional subcategories with > 10 % child-appealing packages (e.g. toaster pastries, hot cocoa and shakes) were also included in the sample (Table 1). To enable direct comparison of the proportion of products with and without CAP across all priority categories, these subcategories were retained regardless of the number or prevalence of child-appealing products. The final analytical sample included 5850 products of which products with child-appealing marketing (e.g. characters, games and fun designs) were also identified from FLIP 2017^(27)^.

**Table 1.** Description of food subcategories used for analysis^(27)^

Food Category Name	Number of products	TRA subcategories included*	Description of included products*
Cakes	155	A6	Medium weight cake, such as cake with or without icing or filling, cake with less than 35 % of the finished weight as fruit, nuts or vegetables or any of these combined; light weight cake with icing; Boston cream pie, cupcakes, eclairs, or cream puffs, individually wrapped snack cakes such as Swiss rolls
Candy	573	U1, U11, U10	Candies, confectionaries and chocolates, including a mixture of these with or without other food added, chocolate bars and other chocolate products, Halloween candy, box of assorted chocolates, Marshmallows; fruit leather, bar or mini pieces, which may or may not contain vegetable ingredients
Cereal	88	C3	Ready-to-eat breakfast cereals, puffed and coated, flaked, extruded, without fruit or nuts (weighing 20 g to 42 g per 250 ml), very high fibre cereals (with 28 g or more fibre per 100 g)
Cheese	569	D1	Cheese, including cream cheese and cheese spread
Cookies	523	A10	Cookies, with or without coating or filling; graham wafers
Crackers	66	A12	Snack crackers, crackers and cheese dip pack
Drinkable Yogurt	60	D12	Fermented dairy drinks including drinkable yogurts, kefir
Fruit Sauce	65	J6	Apple (or other fruit) sauces, including those that contain other fruit and vegetables
Grain bars	199	A18, A19	Grain-based bars with or without filling or partial or full coating
Hot Cocoa	32	B5	Cocoa and chocolate beverages (hot)
Ice cream	499	E1, E2, E3, E4	Ice cream, ice milk, frozen yogurt, sherbet, other frozen dairy and non-dairy desserts sold in tubs, cakes, sandwiches, cones, pops, bars or cups, including sorbet and gelato; Sundaes
Juice	608	J11	Juices, nectars and fruit drinks represented for use as substitutes for fruit juices, juice-based smoothies
Meals	1012	N1, N2	Combination dishes, such as macaroni and cheese, spaghetti with sauce, lasagna, stir fry, casserole, wieners and beans, chili, ravioli with sauce, beef stroganoff, poultry à la king, stew, poutine, butter chicken with rice, shepherd’s pie, burritos, pizza, pastry rolls, quiche, sandwiches, crackers and meat or poultry lunch-type packages, burger on a bun, frank on a bun, calzones, tacos, pockets stuffed with meat, empanadas, fajitas, sushi, souvlaki, pot pie
Meats	214	L7	Patties, meatballs, sausage meat and ground meat, with or without breading or batter, corn dog on a stick (breaded), falafels, including simulated meat and poultry products
Milk	201	D11	Milk, buttermilk and milk-based drinks, such as chocolate milk, plant-based beverages
Nut Butter	101	O3	Peanut butter, nut butters and substitutes, such as coconut and soya butter
Pudding	180	E5	Custard, gelatin and pudding
Shakes	25	D13	Shakes, with or without coffee or juice, including protein shakes and dairy substitute shakes, smoothies (if whey/dairy or plant-based beverage is a main ingredient), milkshakes
Snacks	577	S1	Chips, pretzels, popcorn, extruded snacks, grain and pulse-based snacks, pita chips and fruit-based snacks, such as fruit chips
Syrups/Spreads	92	U8, U15	Honey, molasses and bread spreads; syrups used as ingredients, such as corn syrup, agave syrup and flavoured syrups for milk
Toaster Pastries	11	A14	Toaster pastries

TRA, table of reference amounts for food. *Subcategories and descriptions adapted from Health Canada’s Table of Reference Amounts for Food, available from: https://www.canada.ca/en/health-canada/services/technical-documents-labelling-requirements/table-reference-amounts-food/nutrition-labelling.html.

The presence of child-appealing marketing was previously identified in this sample using the validated CAP coding tool, described in detail elsewhere^(22)^. Briefly, the CAP tool measures child-appealing marketing on food packaging, based on the marketing techniques. The CAP tool identifies the presence of child-appealing marketing, the type of marketing techniques displayed and the marketing power score based on the total number of techniques displayed. Packaging was manually reviewed by trained research assistants, who assessed all sides of each product’s packaging for predefined marketing techniques based on a published inventory of marketing strategies^(22)^. Packages displaying one or more techniques were classified as CAP. The CAP tool underwent and demonstrated both content and criterion validity in a mixed-methods trial with children^(22)^. The CAP tool was applied to all products in the analytic sample by assessing marketing techniques on all sides of packaging to identify the products with CAP (*n* 746) from the final analytical sample.

### Canadian front-of-package labelling regulations

All products were evaluated against the thresholds for FOP labelling regulations mandated by Health Canada in Canada Gazette II and updated in 2024 to expand the exemptions for dairy products that are good sources of calcium^(12,30)^. The Canadian FOP labelling regulations mandate that all pre-packaged foods meeting and/or exceeding thresholds for sodium, total sugars and/or saturated fat (i.e. nutrients-of-concern) display a ‘high-in’ FOP nutrition symbol. Products are assessed on a per-nutrient basis, and the nutrition symbol would display however many nutrients the product is ‘high in’. The thresholds are set based on the percent daily value (%DV) per stated serving size or reference amounts, whichever is greater, for each nutrient, reference amount, and for two different age groups: standard products (i.e. for adults and children ≥ 4 years of age) or products intended for children one year of age or older but less than four years of age (e.g. toddler food). For the current analysis, thresholds for standard products were applied, as most items in the dataset are general packaged foods rather than toddler-specific products. Table 2 shows the thresholds (%DV and absolute amount per nutrient) used to identify products ‘high in’ nutrients-of-concern according to the regulations. Most prepackaged foods (reference amount or serving size of > 30 g) are assessed for the nutrients-of-concern at the 15 % DV threshold. Whereas foods with a smaller reference amount or serving size (≤ 30 g) and foods that are main dishes with a greater reference amount (≥ 200 g) are subject to 10 % and 30 % DV thresholds, respectively. For main dish products intended solely for children 1–4 years of age, products with a reference amount of 170 g or more are subject to 30 % DV thresholds. Additionally, the regulations include three types of exemptions: (i) health-related exemptions for foods that have shown health benefits (e.g. fruits and vegetables, oils high in unsaturated fats) – this was further extended to include some dairy products that are good sources of calcium^(30)^; (ii) technical exemptions for foods that are not required to display an NFt (e.g. raw single ingredient meats) and (iii) practical exemptions for foods that are well-known sources of nutrients-of-concern (e.g. honey, butter and table salt), whereby if a product meets any of these criteria, it is exempt from the assessment and will not display a nutrition symbol regardless of its levels of nutrients-of-concern.

**Table 2.** Summary of ‘high in’ thresholds for saturated fats, sugars and sodium, according to Health Canada’s FOP labelling regulations^(12)^

Age group	Children 1–4 years			Adults and children ≥ 4 years		
Reference amount	≤ 30 g (or ml)	> 30 g (or ml)	≥ 170 g	≤ 30 g (or ml)	> 30 g (or ml)	≥ 200 g
Thresholds %DV	10 %	15 %	30 %	10 %	15 %	30 %
Amount						
Saturated fat (g)	1	1·5	3	2	3	6
Sugars (g)	5	8	15	10	15	30
Sodium (mg)	120	180	360	230	350	690

FOP, front-of-package; %DV, percent daily value.

### Statistical analysis

The number and proportion of products with CAP and non-CAP that would or would not display a ‘high in’ nutrition symbol was calculated. The proportion of products meeting or exceeding each individual FOP ‘high in’ nutrient threshold (i.e. total sugars, sodium and saturated fat) and number of thresholds (i.e. exempted, 0, 1, 2 or 3) was analysed overall and by TRA subcategory. The difference between the proportion of products that would display a ‘high in’ FOP nutrition symbol between products with CAP and non-CAP was assessed using Fisher’s exact test due to low expected values in many food categories. All statistical analyses were conducted using R Version 4.2.2.

## Results

### Proportion of packaged products with child-appealing packaging

12·8 % (*n* 747/5850) of products in the analytic sample had CAP (Table 3). The categories with the highest proportion of products with CAP were toaster pastries (*n* 11, 100 %), crackers (*n* 28, 42·4 %), cereal (*n* 47, 53·4 %), candy (*n* 151, 26·4 %) and fruit sauce (*n* 17, 26·2 %). Categories with the largest number of products featuring CAP were candy (*n* 151, 26·4 %), ice cream (*n* 99, 19·8 %), cookies (*n* 84, 16·1 %), meals (*n* 66, 6·5 %) and juice (*n* 65, 9·2 %).

**Table 3.** A comparison of the number and proportion of packaged products that would display a ‘high in’ nutrition symbol between those with CAP and non-CAP, according to Health Canada’s FOP labelling regulations

Food category^†^	Total *n*	CAP*						Non-CAP						*P*-value^||^
		*n* ^‡^	%	‘High in’ FOP nutrition symbol^§^		No ‘high in’ FOP nutrition symbol^§^		*n* ^‡^	%	‘High in’ FOP nutrition symbol^§^		No ‘high in’ FOP nutrition symbol^§^		
				*n*	%	*n*	%			*n*	%	*n*	%	
Cakes	155	10	6·5 %	10	100 %	0		145	93·5 %	145	100 %	0		NA
Candy	573	151	26·4 %	149	98·7 %	2	1·3 %	422	73·6 %	417	98·8 %	5	1·2 %	1
Cereal	88	47	53·4 %	24	51·1 %	23	48·9 %	41	46·6 %	6	14·6 %	35	85·4 %	<0·001
Cheese	569	11	1·9 %	0		11	100 %	558	98·1 %	19	3·4 %	539	96·6 %	1
Cookies	523	84	16·1 %	62	73·8 %	22	26·2 %	439	83·9 %	373	85 %	66	15 %	0·02
Crackers	66	28	42·4 %	20	71·4 %	8	28·6 %	38	57·6 %	24	63·2 %	14	36·8 %	0·60
Drinkable Yogurt	60	6	10 %	0		6	100 %	54	90 %	1	1·85 %	53	98·15 %	1
Fruit Sauce	65	17	26·2 %	6	35·3 %	11	64·7 %	48	73·8 %	13	27·1 %	35	72·9 %	0·55
Grain Bars	199	30	15·1 %	9	30 %	21	70 %	169	84·9 %	56	33·1 %	113	66·9 %	0·83
Hot Cocoa	32	1	3·1 %	1	100 %	0		31	96·9 %	31	100 %	0		NA
Ice Cream	499	99	19·8 %	77	77·8 %	22	22·2 %	400	80·2 %	372	93 %	28	7 %	<0·001
Juice	608	56	9·2 %	49	87·5 %	7	12·5 %	552	90·8 %	504	91·3 %	48	8·7 %	0·33
Meals	1012	66	6·5 %	66	100 %	0		946	93·5 %	768	81·2 %	178	18·8 %	<0·001
Meats	214	7	3·3 %	7	100 %	0		207	96·7 %	151	72·9 %	56	27·1 %	0·19
Milk	201	18	9 %	14	77·8 %	4	22·2 %	183	91 %	41	22·4 %	142	77·6 %	<0·001
Nut Butter	101	23	22·8 %	6	26·1 %	17	73·9 %	78	77·2 %	7	9 %	71	91 %	0·07
Pudding	180	17	9·4 %	12	70·6 %	5	29·4 %	163	90·6 %	140	85·9 %	23	14·1 %	0·15
Shakes	25	7	28 %	7	100 %	0		18	72 %	11	61·1 %	7	38·9 %	0·13
Snacks	577	38	6·6 %	29	76·3 %	9	23·7 %	539	93·4 %	261^||^	48·4 %	278	51·6 %	0·001
Syrups/Spreads	92	19	20·7 %	1	5·3 %	18	94·7 %	73	79·3 %	12	16·4 %	61	83·6 %	0·29
Toaster Pastries	11	11	100 %	6	54·5 %	5	45·5 %	0	0 %	0		0		NA
Overall	5850	746	12·8 %	555	74·4 %	191	25·6 %	5104	87·2 %	3352	65·7 %	1752·	34·3 %	<0·001

CAP, child-appealing packaging; FOP, front-of-package. Products included in each category are described in Table 1. Food and beverage products in select categories from University of Toronto’s Food Label Information and Price 2017 database were assessed according to Health Canada’s FOP labelling regulations^(12)^. Products that met either one of the three exemption criteria included in the regulations were exempted: (i) health-related exemptions for foods that have shown health benefits (e.g. fruits and vegetables, oils high in unsaturated fats); this was further extended to include some dairy products that are good sources of calcium^(30)^; (ii) technical exemptions for foods that are not required to display a NFt (e.g. raw single ingredient meats) and (iii) practical exemptions for foods that are well-known sources of nutrients-of-concern (e.g. honey, butter and table salt), whereby if a product meets any of these criteria, they do not need to display an FOP nutrition symbol. Any products not exempted from the regulations were assessed for levels of nutrients-of-concern (saturated fat, total sugars and sodium) of percent daily value (%DV) thresholds, set based on the target age group and the reference amount (as per Health Canada’s Table of Reference Amounts for Foods (TRA)^(26)^. Products meeting or exceeding any of the thresholds of nutrients-of-concern would be required to display a ‘high in’ FOP nutrition symbol. “No ‘high-in’” refers to products that are exempt or do not meet or exceed any of the nutrient thresholds. *The CAP coding tool was used to identify products with CAP within the sample^(22)^; ^†^food categories making up the top 85 % of child-appealing packages were selected^(27)^. Any additional categories with > 10 % CAP were added to the sample; ^‡^number and percentage of total sample size; ^§^percentage of total products analysed in that food category, in that analytic sample. ^||^Difference between the proportion of products that would display a ‘high in’ FOP nutrition symbol between products with CAP and non-CAP was assessed using Fisher’s exact test due to low expected values in many food categories. *P* value < 0·05 was considered significant.

### Proportion of packaged products that would display Canada’s ‘high in’ front-of-package nutrition symbol

Overall, 66·8 % (*n* 3907) of products in the analytical sample would display a ‘high-in’ FOP nutrition symbol based on Canada’s FOP labelling regulations (Table 3). The categories with the highest proportion of products that would display a nutrition symbol were cakes (100 %, *n* 155/155), hot cocoa (100 %, *n* 32/32) and candy (98·8 %, *n* 566/573). More than three-quarters of products in iuice (91 %, *n* 553/608), ice cream (90 %, *n* 449/499), pudding (84·4 %, *n* 152/180), cookies (83·2 %, *n* 435/523) and meals (82·4 %, *n* 834/1012) would also display a nutrition symbol. Categories with the lowest proportion of products that would display a nutrition symbol were those that qualified for exemptions, including the updated expansion of dairy-related exemptions: Drinkable yogurt (1·7 %, *n* 1/60), cheese (3·3 %, *n* 19/569), nut butter (12·9 %, *n* 13/101), syrups/spreads (14·1 %, *n* 13/92) and milk (27·4 %, *n* 55/201).

### Comparison of the proportion of packaged products that would display Canada’s ‘high in’ nutrition symbol between products with child-appealing packaging and child-appealing packaging

Overall, the proportion of products with CAP that would display a ‘high in’ FOP nutrition symbol was higher than that of products with non-CAP products (74·4 % *v*. 65·7 %, respectively, *P* < 0·001) (Table 3). Across products in the cereal (51·1 % *v*. 14·6 %, *P* < 0·001), meals (100 % *v*. 81·2 %, *P* < 0·001), milk (77·8 % *v*. 22·4 %, *P* < 0·001) and snacks (76·3 % *v*. 48·4 %, *P* < 0·001) categories, a significantly greater proportion of products with CAP would display a nutrition symbol compared with products with non-CAP (Table 3). In contrast, there was a significantly higher proportion of products with non-CAP in the cookies (85 % *v*. 73·8 %, *P* = 0·02) and ice cream (93 % *v*. 77·8 %, *P* < 0·001) categories that would display a nutrition symbol compared with products with CAP.

### Comparison of the proportion of packaged products meeting 0–3 nutrient thresholds between products with child-appealing packaging and non-child-appealing packaging

Of the products with CAP (*n* 746), 5·4% (*n* 40) would be exempted from the FOP labelling regulations, 20·2% (*n* 151) would not display a ‘high in’ FOP nutrition symbol, 49·1% (*n* 366) would display a symbol for being ‘high in’ one nutrient of concern, 24·8% (*n* 185) for being ‘high in’ two nutrients and 0·5% (*n* 4) for being ‘high in’ all three nutrients (Table 4). Of the products with non-CAP (*n* 5104), 14·5 % (*n* 741) would be exempted from the FOP labelling regulations, 19·8% (*n* 1011) would not display a nutrition symbol, 36·8% (*n* 1879) would display a symbol for being ‘high in’ one nutrient-of-concern, 27·4% (*n* 1399) for being ‘high in’ two nutrients and 1·4% (*n* 74) for being ‘high in’ all three nutrients (Table 4).

**Table 4.** Proportion of products with CAP *v*. non-CAP meeting or exceeding 0–3 nutrient thresholds based on Canada’s FOP labelling regulations

Food category^†^	CAP*											Non-CAP										
	*n*	No ‘high in’ FOP nutrition symbol^‡^				‘High in’ FOP nutrition symbol^‡^						*n*	No ‘high in’ FOP nutrition symbol^‡^				‘High in’ FOP nutrition symbol^‡^					
		Exempted		No. ≥ thresholds									Exempted		No. ≥ thresholds							
				0		1		2		3					0		1		2		3	
		*n*	%	*n*	%	*n*	%	*n*	%	*n*	%		*n*	%	*n*	%	*n*	%	*n*	%	*n*	%
Cakes	10	0		0		0		9	90 %	1	10 %	145	0		0		20	13·8 %	99	68·3 %	26	17·9 %
Candy	151	0		2	1·3 %	119	78·8 %	30	19·9 %	0		422	0		5	1·2 %	157	37·2 %	260	61·6 %	0	
Cereal	47	0		23	48·9 %	22	46·8 %	2	4·3 %	0		41	0		35	85·4 %	6	14·6 %	0		0	
Cheese	11	11	100 %	0		0		0		0		558	501	89·8 %	38	6·8 %	12	2·2 %	7	1·3 %	0	
Cookies	84	0		22	26·2 %	36	42·9 %	26	31 %	0		439	0		66	15 %	155	35·3 %	218	49·7 %	0	
Crackers	28	0		8	28·6 %	18	64·3 %	2	7·1 %	0		38	0		14	36·8 %	22	57·9 %	2	5·3 %	0	
Drinkable Yogurt	6	6	100 %	0		0		0		0		54	52	96·3 %	1	1·9 %	1	1·9 %	0		0	
Fruit Sauce	17	2	11·8 %	9	52·9 %	6	35·3 %	0		0		48	14	29·2 %	21	43·8 %	13	27·1 %	0		0	
Grain Bars	30	0		21	70 %	7	23·3 %	2	6·7 %	0		169	0		113	66·9 %	38	22·5 %	18	10·7 %	0	
Hot Cocoa	1	0		0		0		1	100 %	0		31	0		0		2	6·5 %	6	19·4 %	23	74·2 %
Ice Cream	99	0		22	22·2 %	16	16·2 %	61	61·6 %	0		400	0		28	7 %	103	25·8 %	266	66·5 %	3	0·8 %
Juice	56	0		7	12·5 %	49	87·5 %	0		0		552	0		48	8·7 %	500	90·6 %	4	0·7 %	0	
Meals	66	0		0		28	42·4 %	38	57·6 %	0		946	0		178	18·8 %	428	45·2 %	331	35 %	9	1 %
Meats	7	0		0		7	100 %	0		0		207	20	9·7 %	36	17·4 %	80	38·6 %	71	34·3 %	0	
Milk	18	1	5·6 %	3	16·7 %	11	61·1 %	0		3	16·7 %	183	58	31·7 %	84	45·9 %	36	19·7 %	2	1·1 %	3	1·6 %
Nut Butter	23	2	8·7 %	15	65·2 %	6	26·1 %	0		0		78	35	44·9 %	36	46·2 %	6	7·7 %	1	1·3 %	0	
Pudding	17	0		5	29·4 %	12	70·6 %	0		0		163	0		23	14·1 %	74	45·4 %	62	38 %	4	2·5 %
Shakes	7	0		0		1	14·3 %	6	85·7 %	0		18	0		7	38·9 %	10	55·6 %	0		1	5·6 %
Snacks	38	0		9	23·7 %	22	57·9 %	7	18·4 %	0		539	0		278	51·6 %	212	39·3 %	44	8·2 %	5	0·9 %
Syrups/Spreads	19	18	94·7 %	0		0		1	5·3 %	0		73	61	83·6 %	0		4	5·5 %	8	11 %	0	
Toaster Pastries	11	0		5	45·5 %	6	54·5 %	0		0		0	0		0		0		0		0	
Overall	746	40	5·4 %	151	20·2 %	366	49·1 %	185	24·8 %	4	0·5 %	5104	741	14·5 %	1011	19·8 %	1879	36·8 %	1399	27·4 %	74	1·4 %

CAP, child-appealing packaging; FOP, front-of-package. Products included in each category are described in Table 1. Food and beverage products in select categories from University of Toronto’s Food Label Information and Price 2017 database were assessed according to Health Canada’s FOP labelling regulations^(12)^. Products that met either one of the three exemption criteria included in the regulations were exempted: (i) health-related exemptions for foods that have shown health benefits (e.g. fruits and vegetables, oils high in unsaturated fats); this was further extended to include some dairy products that are good sources of calcium^(30)^; (ii) technical exemptions for foods that are not required to display a NFt (e.g. raw single ingredient meats) and (iii) practical exemptions for foods that are well-known sources of nutrients-of-concern (e.g. honey, butter and table salt), whereby if a product meets any of these criteria, they do not need to display an FOP nutrition symbol. Any products not exempted from the regulations were assessed for levels of nutrients-of-concern (saturated fat, total sugars and sodium) of percent daily value (%DV) thresholds set based on the target age group and the reference amount (as per Health Canada’s Table of Reference Amounts for Foods (TRA)^(26)^. Products meeting or exceeding any of the thresholds of nutrients-of-concern would be required to display a ‘high in’ FOP nutrition symbol. “No ‘high-in’” refers to products that are exempt or do not meet or exceed any of the nutrient thresholds. *The CAP coding tool was used to identify products with CAP within the sample^(22)^; ^†^food categories making up the top 85 % of child-appealing packages were selected^(27)^. Any additional categories with > 10 % child-appealing packages were added to the sample; ^‡^percentage of total products analysed in that food category, in that analytic sample.

### Comparison of the proportion of packaged products that would meet or exceed each individual ‘high in’ nutrient threshold between products with child-appealing and non-child-appealing packaging

Over half of the products with CAP were ‘high in’ sugars (54·4 %, *n* 406), 30·6 % (*n* 228) were ‘high in’ saturated fat and 15·3 % (*n* 114) were ‘high in’ sodium (Table 5). 37·8 % (*n* 1931) of products with non-CAP were ‘high in’ sugars, 34·5 % (*n* 1763) were ‘high in’ saturated fat and 23·6 % (*n* 1205) were ‘high in’ sodium (Table 5). Overall, a greater proportion of food products with child appealing marketing were high in sugars compared with those without.

**Table 5.** Proportion of products with CAP*v*. non-CAP that would meet or exceed ‘high in’ nutrient thresholds for saturated fat, sugars and sodium

Food category^†^	Total *n*	CAP*									Non-CAP								
		*n*	No ‘high in’ FOP nutrition symbol^‡^		Nutrient-of-concern type^‡^						*n*	No ‘high in’ FOP nutrition symbol^‡^		Nutrient-of-concern type^‡^					
					Saturated fat		Sugars		Sodium					Saturated fat		Sugars		Sodium	
			*n*	%	*n*	%	*n*	%	*n*	%		*n*	%	*n*	%	*n*	%	*n*	%
Cakes	155	10	0		10	100 %	10	100 %	1	10 %	145	0		131	90·3 %	135	93·1 %	30	20·7 %
Candy	573	151	2	1·3 %	31	20·5 %	148	98 %	0		422	5	1·2 %	341	80·8 %	336	79·6 %	0	
Cereal	88	47	23	48·9 %	0		22	46·8 %	4	8·5 %	41	35	85·4 %	0		2	4·9 %	4	9·8 %
Cheese	569	11	11	100 %	0		0		0		558	539	96·6 %	15	2·7 %	0		11	2 %
Cookies	523	84	22	26·2 %	48	57·1 %	40	47·6 %	0		439	66	15 %	323	73·6 %	268	61 %	0	
Crackers	66	28	8	28·6 %	5	17·9 %	2	7·1 %	15	53·6 %	38	14	36·8 %	3	7·9 %	0		23	60·5 %
Drinkable Yogurt	60	6	6	100 %	0		0		0		54	53	98·15 %	0		1	1·9 %	0	
Fruit Sauce	65	17	11	64·7 %	0		6	40 %	0		48	35	72·9 %	0		13	38·2 %	0	
Grain Bars	199	30	21	70 %	2	6·7 %	9	30 %	0		169	113	66·9 %	30	17·8 %	43	25·4 %	1	0·6 %
Hot Cocoa	32	1	0		1	100 %	1	100 %	0		31	0		28	90·3 %	31	100 %	24	77·4 %
Ice Cream	499	99	22	22·2 %	66	66·7 %	72	72·7 %	0		400	28	7 %	296	74 %	345	86·3 %	3	0·8 %
Juice	608	56	7	12·5 %	0		49	87·5 %	0		552	48	8·7 %	3	0·5 %	503	91·1 %	2	0·4 %
Meals	1012	66	0		31	47 %	7	10·6 %	66	100 %	946	178	18·8 %	348	36·8 %	49	5·2 %	720	76·1 %
Meats	214	7	0		0		0		7	100 %	207	56	27·1 %	92	49·2 %	0		130	70·3 %
Milk	201	18	4	22·2 %	3	75 %	14	87·5 %	3	21·4 %	183	142	77·6 %	14	23 %	31	39·7 %	4	3·3 %
Nut Butter	101	23	17	73·9 %	6	37·5 %	0		0		78	71	91 %	6	25 %	1	2·7 %	1	2·4 %
Pudding	180	17	5	29·4 %	0		12	70·6 %	0		163	23	14·1 %	20	12·3 %	122	74·8 %	68	41·7 %
Shakes	25	7	0		6	85·7 %	7	100 %	0		18	7	38·9 %	1	5·6 %	11	61·1 %	1	5·6 %
Snacks	577	38	9	23·7 %	17	44·7 %	1	2·6 %	18	47·4 %	539	278	51·6 %	100	18·6 %	32	5·9 %	183	34 %
Syrups/Spreads	92	19	18	94·7 %	1	5·3 %	1	5·3 %	0		73	61	83·6 %	12	16·4 %	8	11 %	0	
Toaster Pastries	11	11	5	45·5 %	1	9·1 %	5	45·5 %	0		0	0		0		0		0	
Overall	5850	746	191	25·6 %	228	30·6 %	406	54·4 %	114	15·3 %	5104	1752	34·3 %	1763	34·5 %	1931	37·8 %	1205	23·6 %

CAP, child-appealing packaging; FOP, front-of-package. Products included in each category are described in Table 1. Food and beverage products in select categories from University of Toronto’s Food Label Information and Price 2017 database were assessed according to Health Canada’s FOP labelling regulations^(12)^. Products that met either one of the three exemption criteria included in the regulations were exempted: (i) health-related exemptions for foods that have shown health benefits (e.g. fruits and vegetables, oils high in unsaturated fats); this was further extended to include some dairy products that are good sources of calcium^(30)^; (ii) technical exemptions for foods that are not required to display a NFt (e.g. raw single ingredient meats) and (iii) practical exemptions for foods that are well-known sources of nutrients-of-concern (e.g. honey, butter and table salt), whereby if a product meets any of these criteria, they do not need to display an FOP nutrition symbol. Any products not exempted from the regulations were assessed for levels of nutrients-of-concern (saturated fat, total sugars and sodium) of percent daily value (%DV) thresholds set based on the target age group and the reference amount (as per Health Canada’s Table of Reference Amounts for Foods (TRA)^(26)^. Products meeting or exceeding any of the thresholds of nutrients-of-concern would be required to display a ‘high in’ FOP nutrition symbol. “No ‘high-in’” refers to products that are exempt or do not meet or exceed any of the nutrient thresholds. *The CAP coding tool was used to identify products with CAP within the sample^(22)^; ^†^food categories making up the top 85 % of child-appealing packages were selected^(27)^. Any additional categories with > 10 % child-appealing packages were added to the sample; ^‡^percentage of total products analysed in that food category in that analytic sample.

The top three categories with CAP that would display a nutrition symbol indicating ‘high in’ sugars content were cakes (100 %, *n* 10), shakes (100 %, *n* 7) and hot cocoa (100 %, *n* 1). The top three categories that would display a nutrition symbol indicating ‘high in’ saturated fat were cakes (100 %, *n* 10), hot cocoa (100 %, *n* 1) and shakes (85·7 %, *n* 6). The top three categories that would display a nutrition symbol indicating ‘high in’ sodium were meals (100 %, *n* 66), meats (100 %, *n* 7) and crackers (53·6 %, *n* 15).

## Discussion

The aim of this study was to evaluate the major policy gap in Canada’s mandatory FOP labelling regulations in relation to the presence of CAP. Overall, the findings of this study indicate that close to three-quarters (74·4 %) of child-appealing products would display a ‘high in’ FOP nutrition symbol for at least one nutrient-of-concern. This proportion was significantly higher than that of products without CAP, particularly for cereal, meals, milk and snacks. These results align with previous research noting the elevated levels of saturated fat, sugars and sodium in food products that are marketed to children in Canada^(24,29,31)^ and highlights a troublesome policy gap that needs to be addressed to ensure that products that display ‘high in’ FOP nutrition symbols cannot be marketed to children on packaging.

Of the 5850 products in the analytic sample, 12·8 % (*n* 747) had CAP. Among these, 74·4 % (*n* 555) would exceed FOP labelling thresholds of 10 %, 15 % or 30 % of the daily value, for one or more nutrients-of-concern, depending on the product’s reference amount, as obtained from Health Canada’s TRA^(12)^. It is important to note that these thresholds are considerably less stringent than the proposed restrictions on M2K^(23)^, which set thresholds of 6 % for sodium, 5 % for sugars and 10 % for saturated fat. Despite the more lenient criteria (i.e. higher amounts of nutrients-of-concern are needed to be required to display the FOP symbol), a substantial proportion of products with CAP still exceeds the FOP labelling thresholds, indicating they are significantly high in nutrients-of-concern. A previous evaluation of the Canadian packaged food supply against Health Canada’s proposed restrictions on M2K found that 98 % of products with CAP would be restricted from M2K^(27)^. However, the proposed M2K policy currently does not extend to product packaging, despite this being a top source of children’s exposure to food marketing^(21,22,24)^. Consequently, foods and beverages high in nutrients-of-concern can still be marketed to children via packaging, despite having to display ‘high in’ FOP nutrition symbols, which undermines the potential for both FOP labelling regulations and the proposed restrictions on M2K. There is therefore a need to extend Canada’s proposed M2K restrictions to include food packaging, thereby ensuring public health nutrition policies in Canada (and elsewhere) have synergistic – rather than conflicting – effects on food choices, diet quality and health.

The findings of the present study agree with previous work by our group and others that products with child-appealing marketing tend to be less healthy than non-child appealing products^(17,18,27)^. The proportion of products with CAP that would display a ‘high in’ FOP nutrition symbol was higher than that of products with non-CAP (74·4 % *v*. 65·75 %, respectively, *P* < 0·001). Notably, all products in our sample with CAP from six categories (cakes, hot cocoa, meals, meats and shakes) would display a ‘high in’ FOP symbol. This is concerning not only because of the influence that marketing to children can have on dietary behaviours and patterns combined with the negative impacts of high intakes of these nutrients-of-concern but also because manufacturers may have little incentive to reformulate if M2K is not prohibited with FOP labelling. The effectiveness of FOP labelling in improving diet is in part due to its ability to incentivise manufacturer-driven reformulation^(8)^. Studies indicate that when manufacturers are required to display FOP nutrition labels, they are more likely to reduce the levels of nutrients-of-concern in their products to avoid negative labelling^(9,11)^. For instance, research has demonstrated that mandatory FOP labels, such as the ‘high in’ warning labels used in Chile, have led to significant reductions in sugar and sodium content in various food categories^(11)^. However, reformulation also increased children’s exposure to low-calorie sweeteners^(32)^, as many reformulated products continued to be marketed to children. Although Chile’s policy package already includes M2K restrictions, this experience highlights that even comprehensive policy approaches can have unintended consequences if not continuously monitored and refined. Restricting M2K remains an essential step, but policymakers should remain attentive to potential unintended outcomes and proactively address emerging loopholes exploited by manufacturers, ultimately advancing towards more comprehensive protective measures for children’s food environment.

The proportion of products with CAP that would be required to display one or more ‘high in’ nutrient on the FOP nutrition symbol(s) was significantly higher than that of products with non-CAP for several food categories, but particularly for breakfast cereals (51·1 % *v*. 14·6 %). This may be, at least in part, related to the high prevalence of child-appealing marketing on cereal boxes, as seen in this study (53·4 % of cereals sampled) as in previous research^(27,33)^. Most child-appealing cereals requiring the FOP nutrition symbol exceeded the sugars threshold, which is unsurprising given the high levels of sugars in children’s breakfast cereals in Canada^(34,35)^. While many breakfast cereals in Canada will be required to display a ‘high in’ FOP nutrition symbol, as demonstrated by this study, these products will still be allowed to carry CAP, thereby hindering the effectiveness of the FOP labelling regulations in encouraging children and caregivers to select healthier options. Chile’s Food Labelling and Advertising Law extends to product packaging^(13,36)^, and research has shown a reduction in the prevalence of breakfast cereals with child-appealing marketing before and after the law was implemented^(36)^. Introducing similar marketing regulations in Canada that include product packaging would likely bolster the effectiveness of FOP labelling in fostering healthier food choices by children/caregivers, particularly for products like breakfast cereals that tend to be heavily marketed to children on-package. However, the literature on breakfast cereals is conflicting. Some studies, such as Smith and colleagues’ research analyzing National Health and Nutrition Examination Survey dietary intake data, have reported that children who consumed breakfast cereals had higher diet quality scores compared with those who did not^(37)^. This finding largely reflects the contribution of fortified nutrients (e.g. folic acid, Fe and thiamin) rather than a balanced nutrient profile, as the authors also noted that cereal eaters had significantly higher intakes of total sugars compared with non-eaters^(37)^. Thus, while cereals can provide certain micronutrients, the high sugar content of many children’s cereals remains a significant concern^(34,38)^, and their frequent CAP marketing may give a misleading impression of overall healthfulness. In addition to M2K, limiting other marketing claims as part of FOP labelling regulations could help mitigate these mixed messages for both children and their caregivers.

The findings of the present study and others suggest a need for the Canadian federal government to strongly reconsider incorporating product packaging within the scope of the proposed restrictions on food marketing to children. An alternative option may be to incorporate marketing restrictions into FOP labelling regulations, automatically restricting M2K of products required to bear one or more ‘high in’ FOP nutrition symbols across all traditional and digital media, including packaging, as done in Chile and other countries in the Americas^(39)^. A study evaluating this approach in Chile revealed a significant reduction in the overall prevalence of cereal boxes with CAP following implementation^(36)^. This reduction was primarily due to a decrease in the prevalence of cereals with ‘high in’ FOP labelling using CAP, which dropped from 43 % before implementation to 15 % post-implementation^(36)^. In addition, there will be a need for continued research and monitoring in this area over time as manufacturers and consumers adjust to the implementation of the FOP labelling regulations and potentially, the pending food marketing restrictions proposed by Health Canda.

### Strengths and limitations

To our knowledge, this is the first study to evaluate the policy gap between the recently implemented FOP labelling regulations and the proposed restrictions on M2K in Canada. This work used a large, nationally representative sample of Canadian packaged food and beverage products and included comprehensive comparisons across food categories. This study does, however, have some important limitations. First, this was a cross-sectional analysis at a single point in time, with data collection limited to selected retailers and locations; thus, we may not have captured all products in the sampled food categories that are available in the Canadian food retail marketplace. Similarly, this sample was limited to subcategories that constituted the top 85 % of child-appealing products (as has been done in previous analyses^(27–29)^. Examining the entire FLIP 2017 sample (i.e. all TRA food categories) may have produced slightly different results; for example, the proportion of products in our sample exceeding one or more FOP labelling thresholds (76 %) was considerably higher than that for products in the total FLIP 2017 sample (63·9 %) as indicated by a previous study^(40)^. Furthermore, although some food categories in this analysis contained relatively few products with CAP (e.g. cheeses, hot cocoa and meats), all priority categories were retained to allow consistent comparison across the dataset. While this may limit the statistical generalisability of some category-specific estimates due to small sample sizes, retaining these categories enhances the comprehensiveness of the analysis and supports broader monitoring of potential areas where M2K may emerge in the future. Lastly, this study was based on 2017 data; some products may have since been reformulated, discontinued or newly introduced. While more recent iterations of FLIP (i.e. FLIP 2020) would be more representative of the current food supply, FLIP 2020 was collected via web-scraping due to COVID-19 restrictions on in-store data collection. As a result, we were unable to collect images of all sides of product packaging – particularly the back of package, where child-appealing features like games are often placed. For this study’s research question, which depends on reliable visual identification, FLIP 2017 remains the most appropriate and methodologically complete dataset.

### Conclusions

This study highlights a significant policy gap and inconsistency in Canada’s current nutrition regulations on FOP labelling and proposed M2K restrictions, emphasising the need to extend marketing restrictions to include product packaging and to prohibit products with FOP symbols from M2K. The findings reveal that over three quarters of child-appealing products exceed FOP labelling thresholds for nutrients of concern in 2017 data, which is substantially higher than products without CAP. This discrepancy underscores the urgent necessity for more stringent regulations to prevent the marketing of unhealthy foods to children. To effectively promote healthier food choices, Canada needs to consider incorporating CAP into its proposed food marketing restrictions or automatically restrict the marketing of products that are required to display ‘high in’ FOP nutrition symbols. Continued research and monitoring are essential as these regulations are implemented, ensuring that policies are coherent and complementary to synergistically achieve the intended public health outcomes.
